# Plasmonic Refractive Index and Temperature Sensor Based on Graphene and LiNbO_3_

**DOI:** 10.3390/s22207790

**Published:** 2022-10-14

**Authors:** Muhammad Irfan, Yousuf Khan, Atiq Ur Rehman, Muhammad A. Butt, Svetlana N. Khonina, Nikolay L. Kazanskiy

**Affiliations:** 1 Nanophotonics Research Group, Department of Electronic Engineering, Balochistan University of Information Technology, Engineering and Management Sciences, Quetta 87300, Pakistan; 2 Institute of Microelectronics and Optoelectronics, Warsaw University of Technology, Koszykowa 75, 00-662 Warszawa, Poland; 3 Department of Technical Cybernetics, Samara National Research University, 443086 Samara, Russia; 4 IPSI RAS-Branch of the FSRC “Crystallography and Photonics” RAS, 443001 Samara, Russia

**Keywords:** graphene for sensing, lithium niobate, refractive index sensor, temperature sensor, sensitivity, plasmonic sensor

## Abstract

A high-efficiency dual-purpose plasmonic perfect absorber sensor based on LiNbO_3_ and graphene layers was investigated in this paper for the refractive index and thermal sensing. The sensor design was kept simple for easy fabrication, comprising a LiNbO_3_ substrate with a quartz layer, thin layer of graphene, four gold nanorods, and a nanocavity in each unit cell. The nanocavity is located in the middle of the cell to facilitate the penetration of EM energy to the subsurface layers. The proposed sensor design achieved an output response of 99.9% reflection, which was easy to detect without having any specialized conditions for operability. The performance of the device was numerically investigated for the biomedical refractive index range of 1.33 to 1.40, yielding a sensitivity value of 981 nm/RIU with a figure-of-merit of 61.31 RIU^−1^. By including an additional polydimethylsiloxane polymer functional layer on the top, the device was also tested as a thermal sensor, which yielded a sensitivity level of −0.23 nm/°C.

## 1. Introduction

Lately, the significance of photonic sensors has increased due to the recent pandemic the arises the need to design devices for rapid, efficient, and non-destructive biomedical sensing. Aside from their growing need in biomedicine and health care [1], numeral other fields such as agriculture [2], IoTs [3], and the challenges posed by global warming are pushing sensing technologies to new horizons [4,5]. With each passing day, innovative and more efficient sensing techniques are gaining the attention of researchers ranging from wearable sensors [6] to self-monitoring vital signs of life [7], and meeting the new expectation of the industry 4.0 era [8]. Over the decades, photonic crystal (PhC) based devices such as sensors, filters, waveguides [9,10], and nanowires have proven to be very useful and gradually becoming a major part of the photonics industry. However, localized surface plasmon polariton (LSPP) based sensors have been given close attention due to their highly sensitive nature and the use of new and simplified materials [11,12]. LSPPs have been an integral part of most of the new sensor designs by investing metal layers in the form of gold (Au) or silver (Ag) inside the sensors in a variety of geometrical shapes and structures, thereby proving to be game-changers [13,14]. The high dependency of surface plasmon resonance (SPR) on the sensor perimeter, geometry, and refractive index (RI) of the surrounding medium makes them ideal for sensing applications [15]. Thus far, a countless number of sensors with unique designs have become part of the sensing world.

The research carried out by Ricardo Janeiro et al. [16] proposed a sensor design composed of silicon gratings on a SiO_2_ substrate covered with a polydimethylsiloxane (PDMS) layer operating at around the 1566 nm wavelength range, yielding a sensitivity of 234.8 pm/% for the chemical concentrations and thermal sensitivity of 0.9 nm/°C. On the other hand, in [17], multiple designs of Au rings and split Au rings over a graphene layer with notches were proposed for RI sensing and it was shown that the sensor design was acceptable with a range of applications. However, the design can be complex from the fabrication point of view. In another concept investigated by Zhu et al. [18], plasmonic sensor design on fiber optics tips based on PDMS was experimented and a maximum sensitivity level of −4.13 nm/°C was reported. Similarly, a sensor design comprising Ag nanoparticles [19] was also investigated and tested as the RI sensor with glucose samples showing a maximum sensitivity of 1144 nm/RIU. Nejat et al. [20] claimed a highly sophisticated sensor design by using MIM Ag-air grating with a complex grooved structure and reported a sensitivity as high as 1460 nm/RIU for glucose syrup and other biological fluids. In a few research works [21,22,23,24,25], new and improved designs have been proposed for RI sensing with different levels of complexity and suitability for various practical applications.

The above-discussed sensor designs provide promising sensitivity values with the trade-off between different factors such as design complexity, the use of costly materials, and cost-effectiveness with less-complex designs. This research work presents a compact, easy to fabricate, and cost-effective plasmonic sensor design comprising of a multi-layered structure that operates in the visible spectral range between 400 and 500 nm. The multi-layer design is comprised of lithium niobate (LiNbO_3_) layer, quartz glass, graphene, Au rings, and a PDMS functional layer, making up a compact design with a 400 nm perimeter and 260 nm height. The materials used are quite economical and the response window is easily tunable, depending on the thickness of the incorporated layers or by scaling the geometry of the overall sensor design. In sensor design, the material cost is reduced by introducing Au cylinders that are deposited in a comparatively lesser quantity, only necessary to achieve plasmonic properties. As a crucial replacement, the same can also be investigated with Ag or other suitable plasmonic materials to decrease the cost even further. Au and Ag are often taken as comparatively better plasmonic candidates than other metals due to their oxidation resistance characteristics [26]. This design proposes both rods and holes—often used alternatively in the majority of sensor designs—for obtaining a sharper sensor response in terms of figure of merit (FOM) and sensitivity. The Au rods coupled with a dielectric layer, is stacked with an adjacent graphene layer to enhance the energy coupling between the two surfaces. Additionally, this paper presents LiNbO_3_ as a potential key material, acting as the base foundation for plasmonic sensors, considered as a comparatively new entry to photonics field, and it was noticed that this material acted better than either of the traditional dielectric materials (i.e., MgF_2,_ and TiO_2_), which are in majority of cases used for waveguiding. The investigated model was designed in CST Studio that incorporates the finite element method (FEM) technique using the tetrahedral meshing for calculations.

## 2. Sensor Design and Materials

The material composition of the sensor was kept simpler for the sake of easier fabrication and characterization. The base was composed of a 200 nm thick LiNbO_3_ layer for better trapping and confinement of the EM energy. Furthermore, it helps to suppress any energy transmission through the model, which is desirable for all perfect absorber RI sensors. Additionally, LiNbO_3_ is ideal for a variety of optical applications considering its outstanding piezoelectric, electro-optic, and nonlinear optical characteristics. Integrated optical devices are increasingly being made on LiNbO_3_ substrates due to its extended transmission range and the absence of residual birefringence. Therefore, it is presumed as a good contender for many applications because of its excellent resistance to thermal expansion and contraction. It is also considered as a good choice for second harmonic generation (SHG) lasers and other optoelectronic devices because of its stability under high-power situations [27,28,29,30]. Since the proposed sensor device is also tested for temperature sensing, therefore, a LiNbO_3_ substrate was selected for more stable and reliable temperature sensing applications.

**Figure 1:**
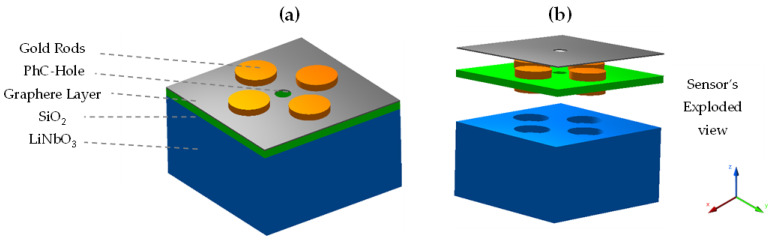
(**a**) Sensor model and (**b**) sensor’s exploded view for the conceptualization of individual layers.

The graphene layer is segregated from the substrate using a 20 nm thick SiO_2_ layer, compulsory for the adherence of contrast materials with each other [31]. The graphene layer is critical for the overall sensor design as it forms a thin and highly conductive surface over the two dielectric layers. Graphene is well-known for its efficiency to detect RI variation over its surface, hence enhancing the overall sensitivity of the sensor. Its thickness was kept at 0.34 nm for an optimum sensor design [32,33]. The four Au nanorods were placed from the top layer down to the LiNbO_3_ foundation layer for creating stronger plasmonic pulses across all adjacent layers. The dimensions of the Au nanorods were chosen to be 50 nm in radius and 60 nm in height. For better EM penetration and the containment of photons within the sub-layers, a nanohole with a 20 nm radius was carved at the center to form a PhC structure. All the geometrical parameters of the sensor model were optimized in the numerical simulations for a range of best-fitting values to enable proper tuning and impedance matching. The simulation model with its exploded view is shown in Figure 1a,b. All of the parametric measurement values of the model design are listed in Table 1.

## 3. Methodology

### 3.1. Modeling and Simulation Presets

**Figure 2:**
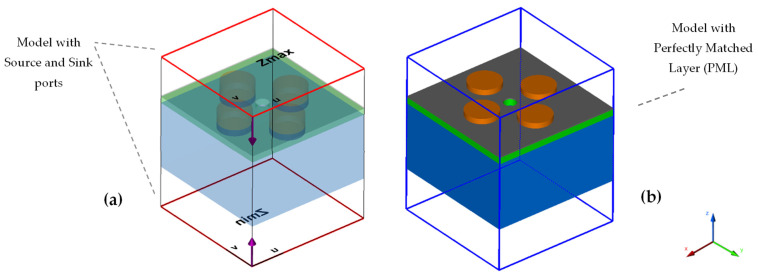
(**a**) Source (*Z*_max_) and sink (*Z*_min_) ports above and below the model. (**b**) Indication of PML boundary to suppress the unwanted EM energy.

The numerical modeling of the sensor was carried out in CST Studio based on the finite element method (FEM) and the tetrahedral meshing technique was used for the calculations. To reduce the simulation time phenomenally in the dense mesh frequency domain technique (which has a higher accuracy over time domain in nanoscale models), the unit cell model technique was used. To terminate the simulation domain, Floquet periodic boundary conditions (PBC) were used in the x- and y-directions and open boundaries were used in the z-direction. Additionally, the perfectly matched layer (PML) condition was used to absorb the unwanted EM field and suppress any higher-order energy modes generated by the periodic structure. Within the boundary of PML, two Floquet-ports named *Z*_max_ and *Z*_min_ were introduced in the z-direction above and below the structure with the background spacing of 100 nm each, so that they could act as a source and sink for the calculation of the reflection and transmission spectra of EM energy in the form of S-parameters. The port setup and PML boundary are indicated in Figure 2a,b.

Only the initial modes of EM and TM were considered in settings in the Floquet ports for both the source and sink layers and the higher order modes were kept unused to save on the simulation time. However, only the electric field focused on the output results for their higher significance and more prominence in the results. The orientation choice was selected as an ‘inward’ direction in the general setup. Furthermore, the Drude–Lorentz model parameters were used for the material properties of the Au ring. As known from fundamental theory, the dispersion properties of Au are linked to the frequency components of the incident EM field described by the Drude–Lorentz model [34]. For the graphene layer, a 0.5 eV potential difference was kept as default.

### 3.2. S-Parameters and the Output Response

The CST ports for calculating the S-parameters for the reflection and transmission spectra of the model were S_11_ and S_21_, respectively, and denoted as in Equations (1) and (2):

(1) $$S_{21}=\frac{\left(1-Z^{2}\right)\Gamma }{1-Z^{2}\Gamma^{2}}$$

(2) $$S_{11}=\frac{\left(1-{\;\Gamma }^{2}\right)Z}{1-Z^{2}\Gamma^{2}}$$

where Z is the impedance parameter and Γ is either the reflection or transmission coefficient depending on the case. The absorbance was later calculated by using Equation (3):

(3) A =1 − T − R

Since the transmission (T) is approximately zero in the case of this sensor (a desirable characteristic), therefore, the absorbance solely relies on reflection. Moreover, in the case of perfect absorption at the time of total energy coupling, the reflectance of the sensor also approaches zero and the absorbance value approaches unity.

## 4. Results and Discussion

The output spectra (transmission/reflection) are commonly monitored based on either the change in the density, wavelength, or phase of the testing material for calculating the sensor’s efficiency [35]. In this case, a shift in wavelength was utilized as a characteristic performance factor. The intermediate values between the biological refractive index range of 1.33 and 1.40 with an increment of 0.01 were set as the input to check the refractive index unit (RIU) sensing of the device. This biomedical index range covers various types of cancer cells, malaria, and different density levels in the human blood. The sensitivity of any RI-based sensor is defined in Equation (4):

(4) $$S=\frac{\delta \lambda }{\delta n}$$

where S is the sensitivity, which is a ratio between the shift in response wavelength of a sensor (δλ) with a change in refractive index (δn) due to sample deposition. On the other hand, the FOM parameter is defined as the S divided by full width at half maximum (FWHM), as stated in Equation (5):

(5) $$\mathrm{Figure}\;\mathrm{of}\;\mathrm{Merit}\;\left(\mathrm{FOM}\right)=\frac{S}{\mathrm{FWHM}}$$

### 4.1. Testing the Device as an RIU Sensor

**Figure 3:**
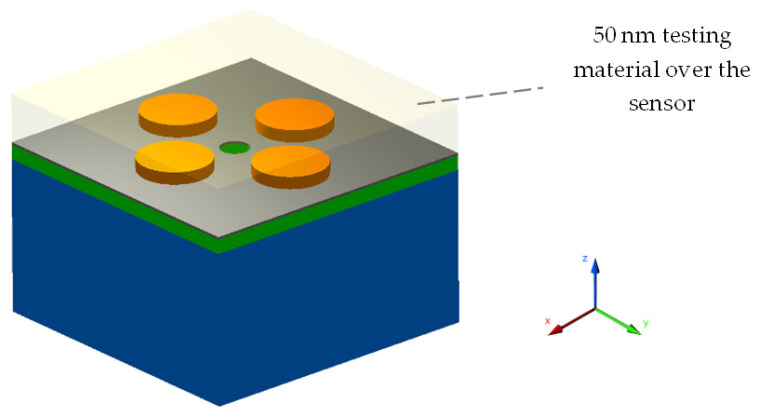
Unit cell design of the sensor with a 50 nm layer of test material deposited on the top.

As a first case study, a 50 nm thick test material was placed on top of the sensor to mimic any real-world solution for RI sensing, as depicted in Figure 3. To evaluate the performance of the sensor, the RI of the test material varied from 1.33 to 1.40.

**Figure 4:**
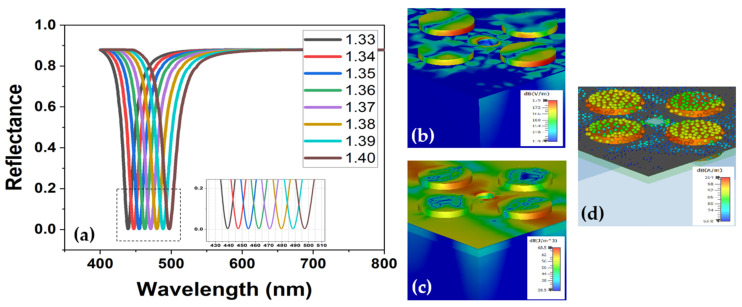
(**a**) Spectral response of the sensor device with reflectance vs. wavelength showing a red shift for variation in RI. (**b**) The E-field distribution showing localization of the EM field on gold nanorods depicting the concentration of surface plasmons. (**c**) The E-field density across sensor design. (**d**) Depiction of the surface current over the metal deposition and graphene layer.

The simulation was performed for each step variation in RI and the value of S was calculated as per Equation (4). In the output spectra illustrated in Figure 4a, it can be observed that a red-shift occurred in the response wavelength of the reflectance (as well as in absorbance) of the sensor. Since the design was optimized to be a perfect absorber, the transmittance is ideally non-existent, and therefore the output response value reached close to unity. Considering the reflectance spectra, the highest shift in the response wavelength was noted as 9.81 nm from 487.45 nm to 497.26 nm for a change in RI from 1.39 to 1.40, which corresponded to a sensitivity value of 981 nm/RIU. The minimum shift in the response wavelength was observed as 7.95 nm from 439.27 nm to 447.22 nm for a RI variation between 1.33 and 1.34.

**Figure 5:**
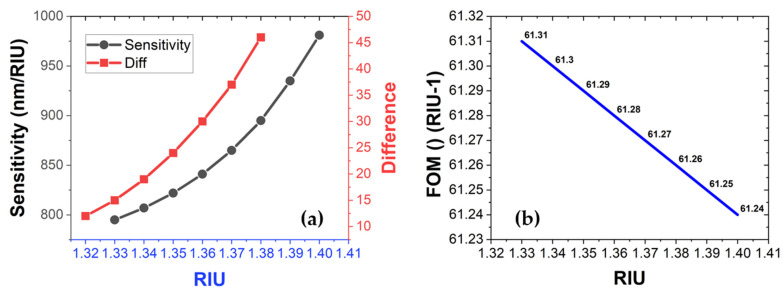
(**a**) Sensitivity vs. RIU for the biological RI range of 1.33 to 1.44. Redline showing the step size of wavelength shift for the tested RI range. (**b**) FOM vs. RIU for the RI range of 1.33 to 1.40.

The proposed sensor design showed a 99.9% absorbance response at the output and therefore, it will require less specialized conditions for practical usage. The right-projection tendency of red-shift response with each deposited sample is understandable because the deposited samples ultimately corresponded to an increase in the surrounding RI of the sensor. It can also be noted that the value of sensitivity increased for higher RI values that corresponded to the shifting of resonant modes to longer wavelengths. Additionally, it is also a known factor for cavity-based sensor designs due to the suppression of low-frequency modes and the enhancement of longer wavelengths [20]. The inset in Figure 4b–d portrays the E-field distribution along each material layer, E-density distribution, and surface current over the structure, respectively. Most of the field energy and current were confined around the conductive surfaces that ultimately triggered plasmons with the adjacent dielectric layers. The noted sensitivity and FOM of the sensor for each RIU variation are summarized in Figure 5a,b. It is conspicuous from both figures that the sensitivity value was higher whereas the FOM was less for longer wavelengths.

### 4.2. Testing the Device as a Temperature Sensor

**Figure 6:**
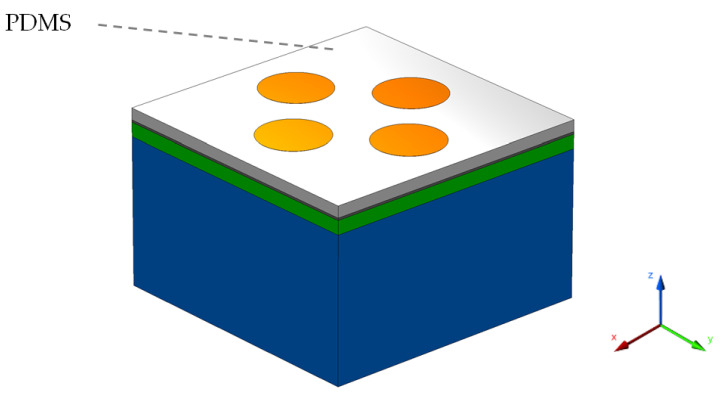
The sensor model showing the presence of the PDMS polymer layer (in white color).

To test the performance of the device as a temperature sensor, the design was slightly modified by the addition of a PDMS polymer layer over the top of the graphene layer up to the level of the Au nanorods as shown in Figure 6. The PDMS material is known for its incompressible and high sensitivity nature to temperature variation. It acts as a transparent material for a wide electromagnetic spectrum except for the infrared range (heat energy). PDMS belongs to the family of siloxane polymers and possesses qualities such as excellent elastic and thermos-optic coefficients with little absorption loss [36]. Therefore, it has been proposed for a variety of biomedical, chemical, and thermal sensing applications. For the sake of simplicity and better control in the proposed device, the PDMS layer was deposited over the top surface rather than inside the design.

**Figure 7:**
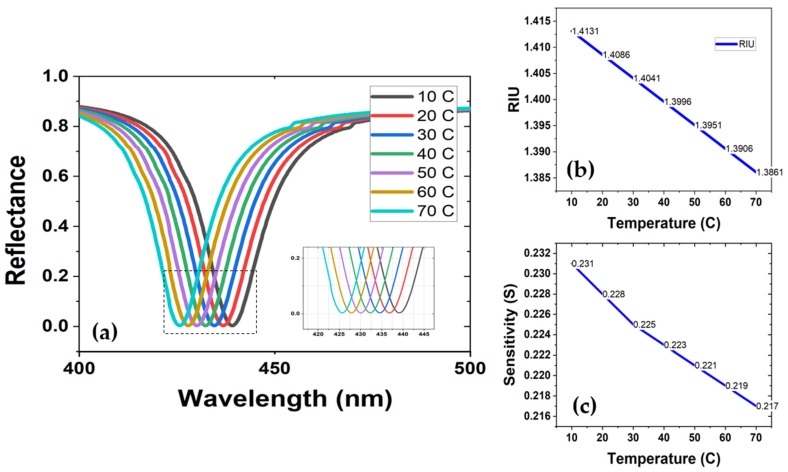
(**a**) Spectral response of the sensor for variation in the ambient temperature, showing a blueshift. (**b**) Temperature vs. RIU. (**c**) Temperature vs. sensitivity.

The sensor design was tested for a range of ambient temperature values from 10 °C to 70 °C. As mentioned earlier, the RI of the PDMS layer changes with the variation in the ambient temperature. The analytical relation between the ambient temperature and variation in the RI of PDMS was taken as a reference from [37] and given by Equation (6). In the numerical simulations, the RI of the PDMS layer was varied to sense the ambient temperature and evaluate the performance of the sensor.

(6) $$\mathrm{RI}\left(\mathrm{PDMS}\right)=1.4176\;–\;4.5\times 10^{-4}\;.\;T$$

where T is the input temperature in degrees Celsius (°C). The equation showed a negative linear relationship between the temperature and the RI, which means that a high temperature will have a lower RI of PDMS and vice versa. Therefore, a blue shift in the output response of the device can be observed, as depicted in Figure 7a. The relation between RI and temperature is shown in Figure 7b.

Considering the performance of the device as a temperature sensor, an average sensitivity value of −0.23 nm/°C was achieved. However, an important aspect of the output response is that the temperature sensitivity was not constant across all of the preset temperature values (10 °C to 70 °C), but differed by a magnitude in accordance and relatively close replication with the linear relationship between the temperature and RI of the PDMS, as shown in Figure 7c. Likewise, the sensitivity values dropped almost linearly as the ambient temperature rose. Table 2 lists the values of RIU, wavelength, and sensitivity that correspond to their respective temperature readings.

### 4.3. Comparative Analysis of the Sensor Design

A comparative analysis of the proposed sensor design for RI and thermal sensing applications with the existing literature is given in Table 3 and Table 4, respectively. It can be seen that the sensor design yielded improved results in comparison to previously reported works in this area. Hence the proposed design serves as a dual-purpose sensor and offers multiple advantage over other designs such as higher sensitivity and FOM values as well as an easy fabrication possibility.

## 5. Proposed Fabrication Steps

**Figure 8:**
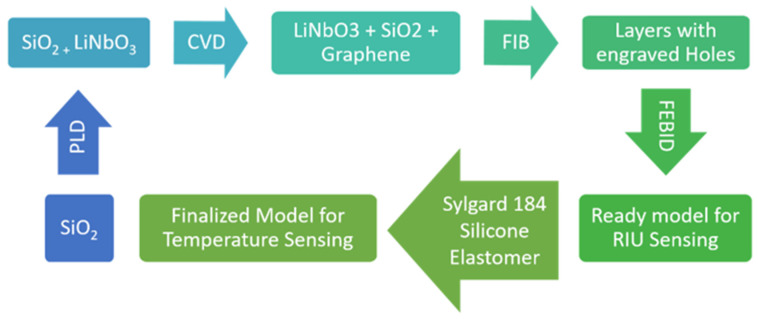
The fabrication process of the proposed sensor design.

The fabrication process of the proposed sensor mainly involves the deposition of thin films, lithography, and etching of the PhC cavity. In the first step, a thin layer of SiO_2_ can be deposited to form the substrate using conventual deposition techniques such as plasma enhanced chemical vapor deposition (PECVD) or ion-beam sputter deposition (IBSD) [44]. In the second step, a LiNbO_3_ layer can be deposited using pulse laser deposition (PLD), which is a modified form of pulse vapor deposition (PVD), giving better accuracy and control over the conventional deposition techniques. In this technique, a high-power pulsed laser beam is focused on a target of the desired material that causes vaporization to deposit a thin film [45]. The twin-layered structure is then inverted and by using the well-known chemical vapor deposition (CVD) method, the thin film of the graphene is added over the SiO_2_ layer. The holes can then be carved using focused ion-beam (FIB) technology with the metal etch-mask technique [46]. As a final stage, focused electron-ion beam induced deposition (FEBID) can be utilized for shaping Au rods over the structure. This last step contributes toward metal deposition and milling to achieve the required shape with the desired characteristics [47]. For the case of temperature sensing, the PDMS layer can be deposited through the direct deposition of sylgard 184 silicon elastomer with heat curing for the proper setting of material. The step-by-step fabrication process is shown in Figure 8. The work conducted in [48] presents the fabrication of a sensor model closely related to the proposed model in this work by using soft nano-imprint lithography. The process involves generating a PDMS stamp through the deposition of silicon on a chromium slab and later using sol–gel resist to form a solid silica model. The PDMS stamp is then used to produce an array of vertical Au nanorods. The structure in the process is annealed multiple times for the settling of the materials. However, the PDMS stamp poses the vulnerability of traced air that deshapes the cylinders and often reduces the heights of the majority of them [49]. Therefore, the PDMS functional layer is proposed to be deposited on top of the sensor at the final stages.

## 6. Conclusions

A plasmonic perfect absorber-based dual sensing device for RI and temperature sensing was numerically investigated in this work. The device is composed of a compact and easy-to-fabricate design with LiNbO_3_ as the base material, a SiO_2_ layer, a graphene layer, Au nanorods, and a PhC-cavity in the middle. The material selection was carefully undertaken to enhance the efficiency of the device. The LiNbO_3_ base enhanced the EM absorption, and graphene was included to improve the sensing capabilities whereas Au nano-rods integrated the plasmonic properties into the design. The device showed a good narrow band filtering performance in the visible spectral range between 400 and 550 nm with almost unity absorption of 99.9%. The design was first tested as a RI sensor for a biological index range of 1.33 to 1.40, which yielded a sensitivity in the range of 981 nm/RIU with a FOM of 61.31 RIU^−1^, which were the highest among the reported similar works. Second, a thin PDMS layer was deposited on the sensor to investigate the device as a thermal sensor for an ambient temperature range of 10 to 70 °C, which yielded a sensitivity in the range of −0.23 nm/°C. Considering its improved design and sensitivity level, the device can be well proposed for experimental implementation and use in a variety of applications ranging from biomedical sensing to chemical laboratories and industrial usage.

## Figures and Tables

**Table 1:** Dimensions of the layers used in sensor design.

Layers (Bottom to Top)	Material	Measurement
Layer 1	LiNbO_3_	200 nm
Layer 2	SiO_2_	20 nm
Layer 3	Graphene	0.34 nm (0.00034 µm)
Layer 4	Gold Nano-rod	50 nm radius × 60 nm L
PhC-hole	Air	20 nm radius
Layer 5	Testing Material or PDMS	50 nm/16.6 nm

**Table 2:** A list of the sensor applications with their associated sensitivity values.

Application	Temperature Range (°C)	Wavelength (nm)	Sensitivity (nm/°C)
Temperature sensing	10	439.27	−0.231
	20	436.96	−0.228
	30	434.68	−0.225
	40	432.43	−0.223
	50	430.20	−0.221
	60	427.99	−0.219
	70	425.80	−0.217

**Table 3:** The RI comparison of the proposed work with the existing literature.

Sensor Design	Sensitivity (nm/RIU)	FOM (RIU^−1^)	Research Work
D-shaped DBR fiber + Au + PDMS	487	-	[38]
Silica spheres + Au	968	2.20	[39]
Periodic Au rings array	557	6.1	[40]
Ag + Si + Ag dual elliptical array	503	63	[41]
Silica + Au slab + Si + Fabry–Perot nanocavities + Au slab	600	28	[42]
LiNb_3_ + graphene	981	61.31	This Work

**Table 4:** The temperature comparison of the proposed work with the existing literature.

Sensor Design	Sensitivity (nm/°C)	Research Work
Sm^+3^:ZnO_2_ (WGM)	0.04	[43]
SMF-28 Silica Fiber	−1.3 *p*m/°C	[44]
Au (slab) + Si (S-MAs & HS-MAs)	−0.18	[37]
LiNb_3_ + Graphene	−0.23	This Work

## Data Availability

Not applicable.
